# A Network Pharmacology Prediction and Molecular Docking-Based Strategy to Explore the Potential Pharmacological Mechanism of *Astragalus membranaceus* for Glioma

**DOI:** 10.3390/ijms242216306

**Published:** 2023-11-14

**Authors:** Yu Feng, Peng Zhu, Dong Wu, Wenbin Deng

**Affiliations:** 1 School of Pharmaceutical Sciences (Shenzhen), Sun Yat-sen University, Shenzhen Campus, Shenzhen 518107, China; fengy83@mail2.sysu.edu.cn; 2 Computer Aided Drug Discovery Center, Zhuhai Institute of Advanced Technology, Chinese Academy of Sciences, Zhuhai 519003, China; zhupeng@ziat.ac.cn

**Keywords:** *Astragalus membranaceus*, glioma, network pharmacology, molecular docking

## Abstract

Glioma treatment in traditional Chinese medicine has a lengthy history. *Astragalus membranaceus*, a traditional Chinese herb that is frequently utilized in therapeutic practice, is a component of many Traditional Chinese Medicine formulas that have been documented to have anti-glioma properties. Uncertainty persists regarding the molecular mechanism behind the therapeutic effects. Based on results from network pharmacology and molecular docking, we thoroughly identified the molecular pathways of *Astragalus membranaceus*’ anti-glioma activities in this study. According to the findings of the enrichment analysis, 14 active compounds and 343 targets were eliminated from the screening process. These targets were mainly found in the pathways in cancer, neuroactive ligand–receptor interaction, protein phosphorylation, inflammatory response, positive regulation of phosphorylation, and inflammatory mediator regulation of Transient Receptor Potential (TRP) channels. The results of molecular docking showed that the active substances isoflavanone and 1,7-Dihydroxy-3,9-dimethoxy pterocarpene have strong binding affinities for the respective targets ESR2 and PTGS2. In accordance with the findings of our investigation, *Astragalus membranaceus* active compounds exhibit a multicomponent and multitarget synergistic therapeutic impact on glioma by actively targeting several targets in various pathways. Additionally, we propose that 1,7-Dihydroxy-3,9-dimethoxy pterocarpene and isoflavanone may be the main active ingredients in the therapy of glioma.

## 1. Introduction

The majority (50–60%) of intracranial tumors are gliomas, which are the most prevalent and aggressive primary malignant tumors of the central nervous system [1,2]. Adults are more likely to experience it, and the frontal and temporal lobes’ white matter is where it frequently manifests. It displays a butterfly-like development pattern and has a significant amount of infiltration. It also frequently crosses the corpus callosum to the opposite side. Gliomas are categorized into grades I through IV based on the histological traits and clinical manifestations, with grades I and II being low-grade malignant gliomas and grades III and IV being high-grade malignant gliomas. Despite the fact that grade I and II gliomas are regarded as low-grade tumors, patients nevertheless need a lengthy course of treatment. Individuals diagnosed with grade III and IV gliomas typically face a high risk of mortality within two years. According to statistics, the incidence of glioma is 2 to 3 cases per 100,000 people. Even with surgical intervention combined with radiotherapy and chemotherapy, the average survival time for patients with low-grade gliomas is only 3 to 5 years, while for high-grade gliomas, it is 1 to 2 years [3].

Most malignancies spread throughout the body and eventually result in death. Gliomas, on the other hand, grow quickly, invade healthy tissues, and are capable of destroying whatever tissue they come into contact with. Gliomas are difficult to locate precisely due to their atypical shape. The two main forms of treatment for gliomas are radiation therapy and surgery, which are frequently combined with chemotherapy. The chemotherapeutic agents temozolomide, nimustine, and avastin are frequently used for treating gliomas [4]. Traditional radiation and chemotherapy techniques frequently cause patients to have severe ill effects, including damage to healthy tissue cells, slowed bone marrow growth, hair loss, gastrointestinal problems, and multidrug resistance.

The natural world has gained popularity in recent years. Traditional Chinese Medicine has drawn a lot of attention because of its comparatively long-lasting benefits and relative safety. In the field of glioma treatment, encouraging advancements have been made, and breakthroughs are anticipated [5]. Unlike Western medicine, Traditional Chinese Medicine treats glioma from a holistic perspective. The general principle of Traditional Chinese Medicine in the treatment of glioma is to strengthen vital qi and eliminate pathogenic qi in the body [6]. Most of the Chinese medicine compound formulas start from the theory system of internal organs. They are primarily employed to combat and eliminate phlegm, blood stasis, and dampness, as well as to cure liver, spleen, and kidney deficiencies. It has special benefits for limiting tumor growth, lowering toxicity, boosting effectiveness, avoiding recurrence, extending longevity, and enhancing quality of life. Many of the Traditional Chinese Medicines and active monomeric components in these Chinese medicine compound formulas have been shown to be effective in treating glioma [6,7,8], such as baicalin [9], ginseng epoxyalkyno [10], saihu saponin [11], burdockin [12], ailanone [13], etc.

Many of the herbal compounds used in the treatment of glioma include *Astragalus membranaceus*, like Shezhihuangqitang (including *Sculellaria barbata*, Spreading hedyotis herb, Smilax Glabra, *Astragalus membranaceus*, *Angelica sinensis*, and Rhubarb), Buyanghuanwutang (including *Astragalus membranaceus*, *Angelica sinensis*, *Paeonia lactiflora Pall*, Earthworm, *Ligusticum sinense*, Persicae Semen, and Safflower), and Xuefuzhuyutang (including *Astragalus membranaceus*, *Persicae Semen*, Safflower, Rehmanniae Radix, Chuanxiong, Red paeony root, Radix Achyranthis Bidentatae, Platycodon grandiflorum, Radix bupleuri, Aurantii Fructus, and Liquorice root). *Astragalus membranaceus* is a traditional Chinese herbal medicine commonly used in clinical practice, which has the effect of correcting and replenishing qi. *Astragalus membranaceus* has been found to have a range of active compounds, as demonstrated by modern pharmaceutical research. These substances include *Astragalus membranaceus* flavonoids, astragtilbene saponins, and polysaccharides [14]. Clinical studies and pharmacological studies have demonstrated *Astragalus membranaceus*’ potent anti-inflammatory [15], immune-modulating [16], anti-aging [17], antiviral [18], and cardiovascular protection [19,20] properties. It has inhibitory effects on the proliferation of liver cancer and lung cancer cells, and can also be used to lower blood glucose levels [21]. The study conducted by Li Zhang et al. suggested that wogonoside, a bioactive flavonoid extracted from the root of *Astragalus membranaceus*, exhibited a cytotoxic effect on human glioblastoma cells. Moreover, the wogonoside-treated cells showed increases in autophagy-related proteins, alterations in cellular shape, and punctate microtubule associated protein 1 light chain 3 (LC3) dots—all of which are hallmarks of autophagy [22]. Research by Xu L et al. showed that *Astragalus membranaceus* IV improves deaminase-induced blood–brain barrier dysfunction by inhibiting the RhoA/Rho-kinase pathway. Although this study did not directly address gliomas, it offers the potential of *Astragalus membranaceus* in improving blood–brain barrier disorders associated with central nervous system tumors [23]. These investigations have demonstrated the potential of *Astragalus membranaceus* to cure gliomas.The diverse and complex composition of *Astragalus membranaceus* makes it difficult to systematically study of the active ingredients in *Astragalus membranaceus* and the molecular mechanism behind its role in anti-glioma needs to be further investigated.

With the development of medical knowledge, disease study is shifting from “reductionist theory” to “systems theory”, and from a single, isolated model to one that has been thoroughly studied from many angles. A considerably important development in this transformation involves the use of a biomolecular network to analyze the relationship between disease and drugs [24]. Network pharmacology, which is based on systems biology, genomics, proteomics and other disciplines, has emerged in recent years. It uses high-throughput omics data analysis, computer simulation and network database retrieval and other technologies to reveal the network relationship of drug–gene–target–disease interaction, and to predict the mechanism of action of drugs through network relationships, evaluate drug efficacy, unhealthy reactions, etc. Network pharmacology’s holistic, systematic, and complete research methodologies make it ideally suited for investigating the several components and multiple targets of traditional Chinese medicine. Network pharmacology has been widely employed in recent years to examine the molecular underpinnings of traditional Chinese medicine [25,26,27].

**Figure 1:**
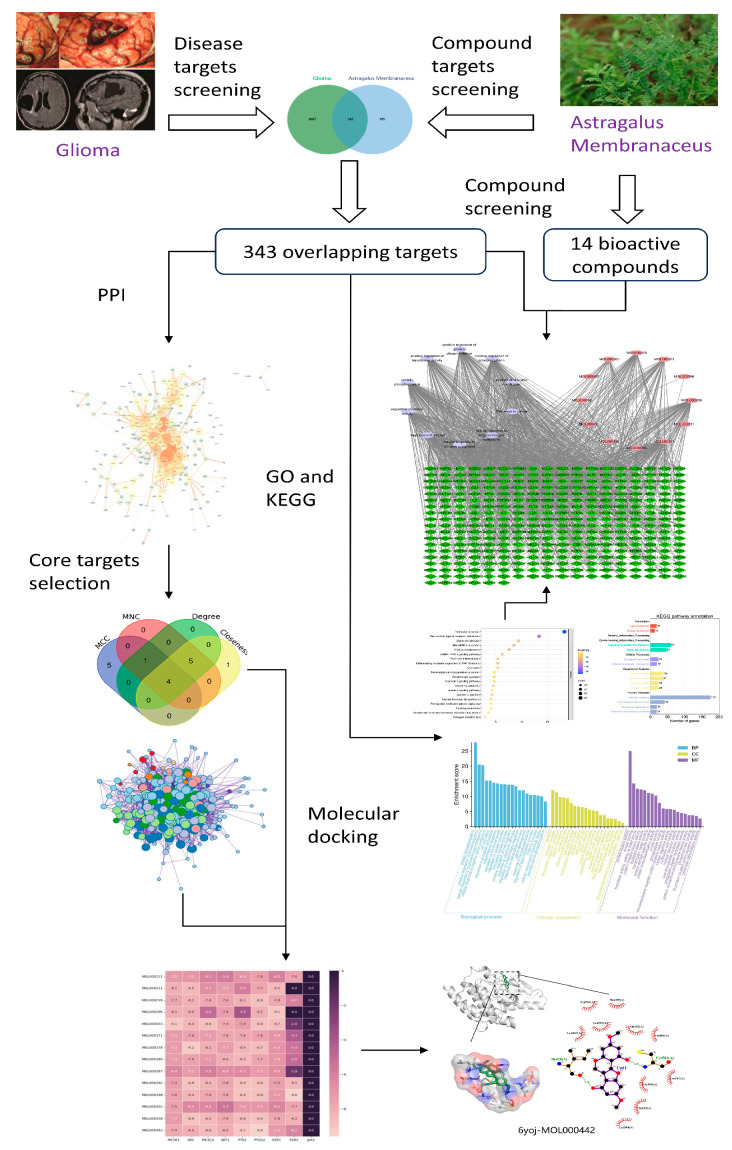
Overall workflow of the study.

In this study, we systematically identified the molecular mechanisms of *Astragalus membranaceus*’ anti-glioma effects based on findings from network pharmacology and molecular docking. Figure 1 displays the study’s flowchart. In this study, the potential targets of *Astragalus membranaceus*’ active components were screened and anticipated, and the targets relevant to glioma were carefully gathered. Then, utilizing network pharmacological approaches, the relationship between medications and targets was investigated. Molecular docking was used to investigate the interaction between the active compounds in *Astragalus membranaceus* and the essential therapeutic targets of glioma. Our research may provide some references and insights for subsequent related studies.

## 2. Results

### 2.1. Active Compounds in Astragalus membranaceus and Their Putative Targets

A total of 14 active compounds were obtained from TCMSP database according to the screening criteria as shown in Table 1. On the one hand, targets for each compound were searched from TCMSP database (Table 1). On the other hand, each compound was predicted to have 100 potential targets through SwissTargetPrediction platform. Targets from both sources were combined and duplicates were removed, resulting in a total of 449 targets.

### 2.2. Targets for Glioma

In total, 10,231, 33,097, and 1350 glioma-related targets were retrieved in GeneCards database, DisGeNET database and BrainBase database, respectively. After combining and deleting duplicate targets from these three disease databases, a total of 88,431 glioma-related targets were obtained.

### 2.3. PPI Network Constructed by the Common Targets

**Figure 2:**
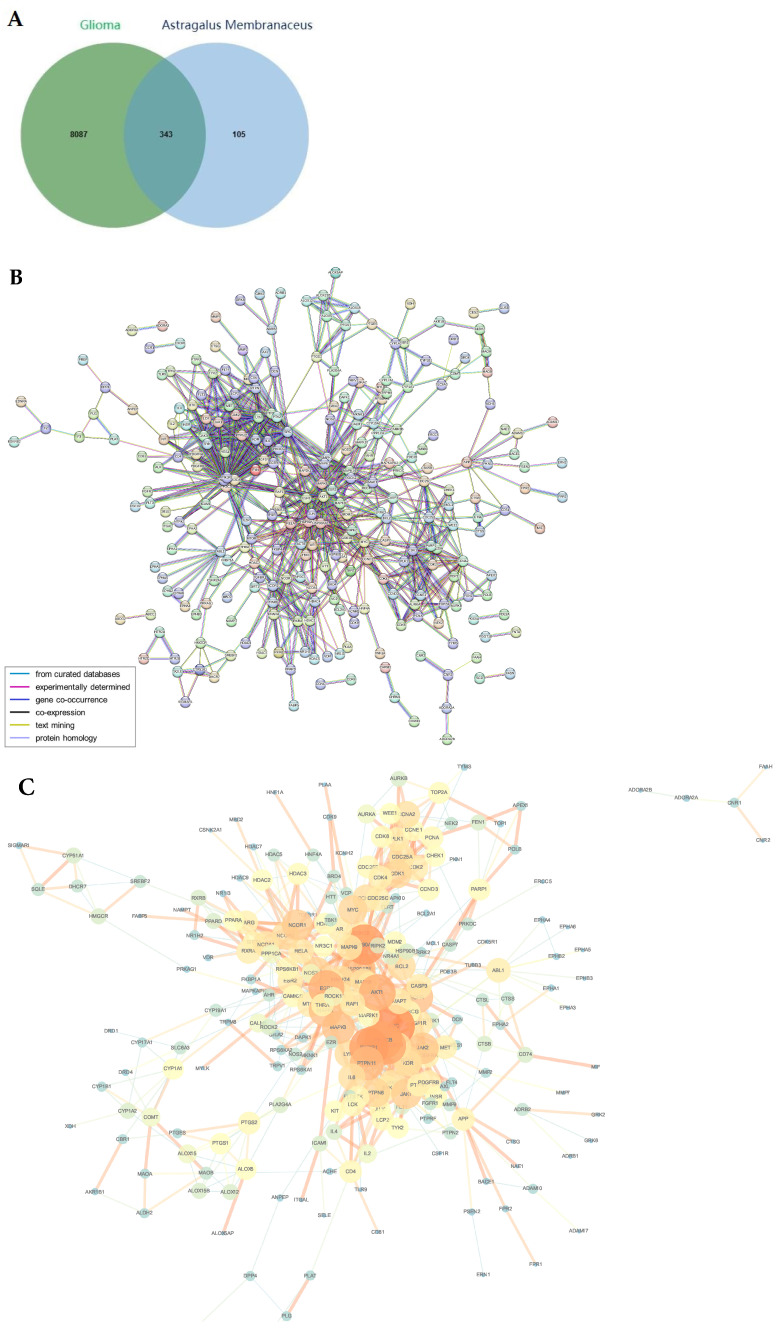
PPI analysis of the intersection targets. (**A**) Venn diagram of the common targets of potential targets of *Astragalus membranaceus* and glioma-related targets. (**B**) PPI network of the 343 intersection targets. (**C**) Topological analysis of the PPI network.

A total of 343 common targets were obtained after Venn mapping of glioma-related disease targets and potential targets of active compounds as shown in Figure 2A. To explore the mechanism underlying the therapeutic effects of active compounds from *Astragalus membranaceus* against glioma, the 343 targets were imported to the STRING database to construct a PPI network. After removing nodes that are not linked to any other nodes, the PPI network consisted of 346 nodes and 902 edges, the average node degree is 5.21, and the local clustering coefficient is 0.465 (Figure 2B). The minimum required interaction score is the highest confidence (0.900). The top ten targets are SRC1 (d = 39), PIK3R11 (d = 37), HSP90AA11 (d = 36), PIK3CA1 (d = 35), PIK3CB1 (d = 35), PIK3CD1 (d = 34), AKT11 (d = 31), ESR11 (d = 31), MAPK11 (d = 27), HSP90AB11 (d = 26). Different colors of lines between edges indicate the type of interaction evidence, as shown in Figure 2B.

An essential Cytoscape tool for thorough network topology study is now NetworkAnalyzer [28]. With Cytoscape, the PPI network was loaded and the NetworkAnalyzer tool was used to analyze the topological network. The density and score of an edge in a network increase with its thickness and the brightness of its color; similarly, a node’s degree and score increase with its size and color. Greater significance in the biological operation of the illness is suggested by larger nodes and brighter hues.

### 2.4. Core Targets Screening and MCODE Module Analysis of the Intersecting Targets

**Figure 3:**
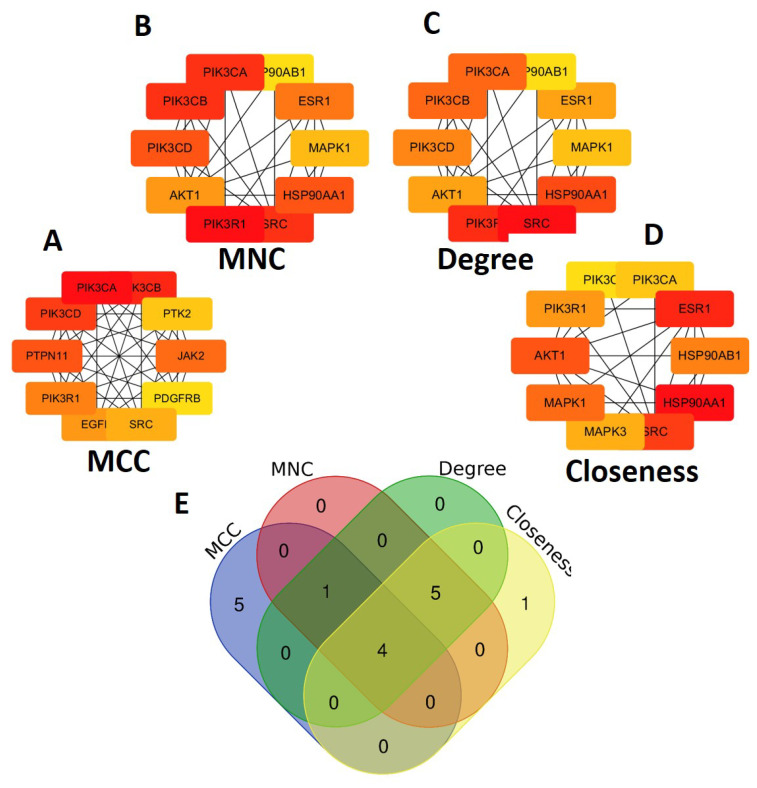
Top 10 core targets identified by (**A**) MCC, (**B**) MNC, (**C**) Degree, and (**D**) Closeness. (**E**) Venn diagram of the common core targets of MCC, MNC, Degree, and Closeness.

**Figure 4:**
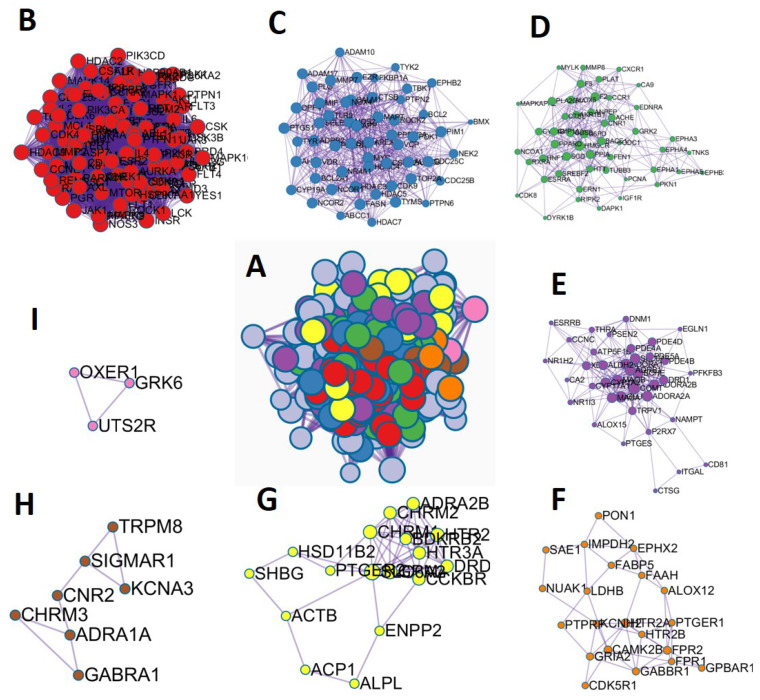
MCODE cluster analysis. (**A**) All MCODE clusters based on 292 intersecting targets. (**B**) Targets cluster for MCODE1. (**C**) Targets cluster for MCODE2. (**D**) Targets cluster for MCODE3. (**E**) Targets cluster for MCODE4. (**F**) Targets cluster for MCODE5. (**G**) Targets cluster for MCODE6. (**H**) Targets cluster for MCODE7. (**I**) Targets cluster for MCODE8.

The CytoHubba [29,30,31] plug-in in Cytoscape was used to identify core targets. MCC algorithm, MNC algorithm, Degree, and Closeness were applied to analyze the hub genes of the intersecting targets of glioma-related disease targets and potential targets of active compounds. Figure 3A and Figure 4D illustrate the top 10 hub genes by each algorithm, and the node’s color changed steadily from pale yellow to red, and so did its degree. Table 2 shows the intersecting genes calculated by different algorithms obtained by Venn analysis (Figure 3E).

Next, using Metascape, the MCODE method was applied to this network to find neighborhoods where proteins are highly related, then eight modules consisting of 292 targets were obtained and visualized by Cytoscape (Figure 4A). As indicated in Figure 4B–I, 100 targets were enriched in MCODE1 (score = 44.25), 56 targets were enriched in MCODE2 (score = 11.16), 50 targets were enriched in MCODE3 (score = 5.62), 38 targets were enriched in MCODE4 (score = 5.5), 21 targets were enriched in MCODE5 (score = 2.19), 17 targets were enriched in MCODE6 (score = 3.05), seven targets were enriched in MCODE7 (score = 1.14), and three targets were enriched in MCODE8 (score = 1). Each MCODE network underwent GO enrichment analysis in order to extract “biological meanings” from the network component, with the top three best *p*-value phrases being kept. Table 3 lists the abundant biological processes of the top three functional modules obtained after enrichment analysis.

### 2.5. GO Function and KEGG Pathways Enrichment Analysis

**Figure 5:**
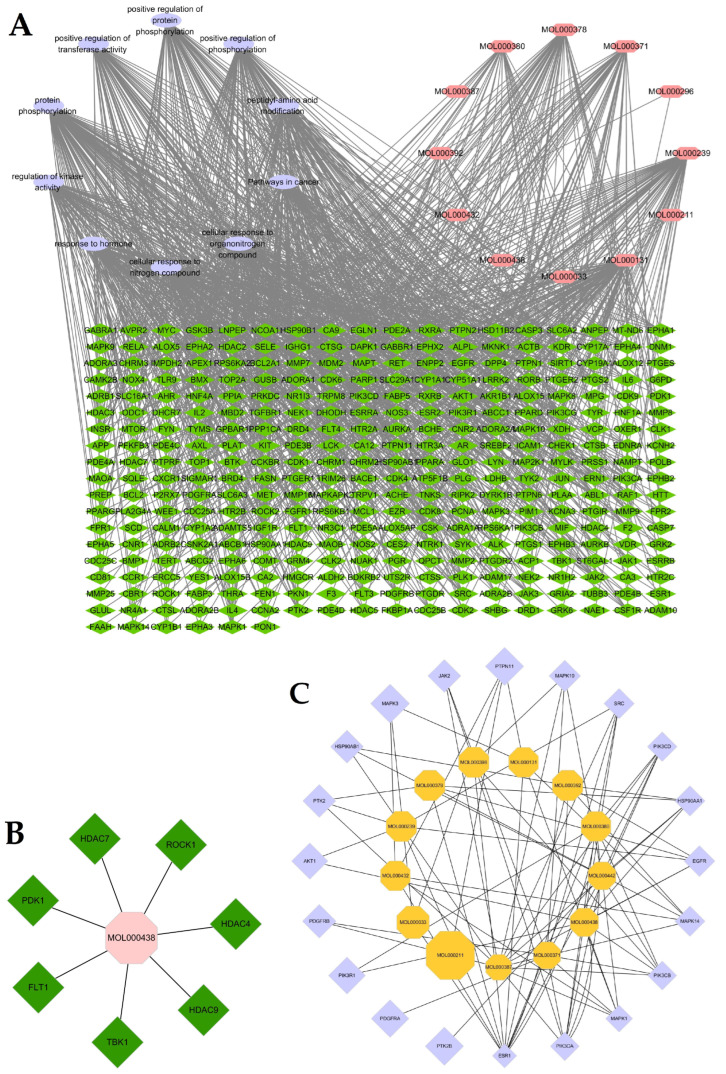
Network construction and analysis. (**A**) Compound–target–pathway network. (**B**) Network of MLO000438 and related targets. (**C**) Compound-core targets network.

To find out which metabolic pathways and biological processes *Astragalus membranaceus* may be able to influence therapeutically in the treatment of glioma, KEGG enrichment and GO functional annotation analyses were performed. A total of 343 common genes were used for enrichment analysis resulting in a total of 2935 GO items, including 164 cellular components (CC) items (Table S1 in the Supplementary Materials), 343 molecular functions (MF) items (Table S2 in the Supplementary Materials), and 2428 biological processes (BP) items (Table S3 in the Supplementary Materials). The top 20 correlation terms for each category were ranked based on the number of annotations to a functional area, and are shown in Figure 5A. Cellular components were mainly distributed in the dendrite, receptor complex, perinuclear region of the cytoplasm, side of membrane and membrane raft. Molecular function mainly involved protein kinase activity, hydrolase activity, protein tyrosine kinase activity, oxidoreductase activity, and transcription factor binding. Biological processes were mainly involved in protein phosphorylation, positive regulation of phosphorylation, cellular response to organonitrogen compounds, and cellular response to organic cyclic compounds.

Furthermore, 215 KEGG pathways (Table S4 in the Supplementary Materials) were identified through enrichment analysis and the top 20 pathways are shown in Figure 5B. The KEGG pathways were mainly enriched in pathways in cancer, neuroactive ligand–receptor interaction, viral carcinogenesis, Alzheimer’s disease, and inflammatory mediator regulation of Transient Receptor Potential (TRP) channels.

**Figure 6:**
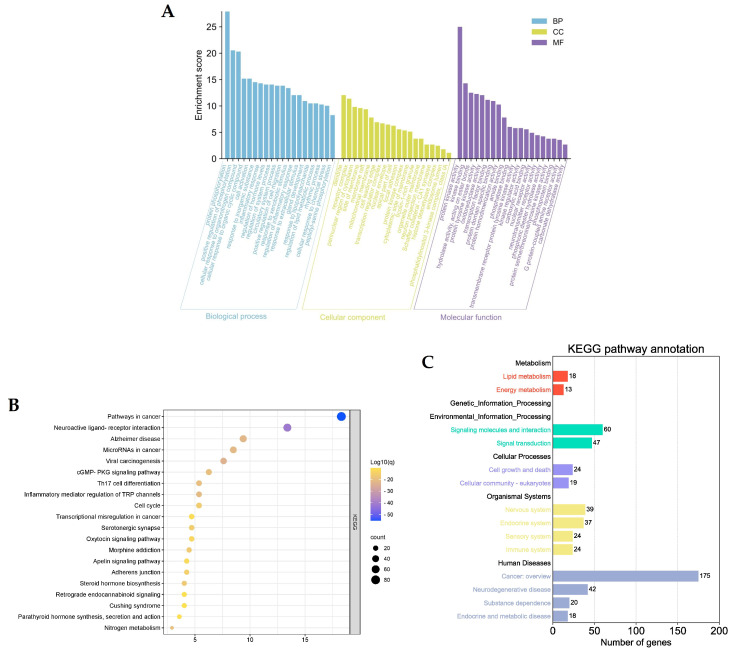
GO function and KEGG pathways enrichment analysis of 343 intersecting targets. (**A**) GO function analysis. (**B**) KEGG pathways enrichment analysis. (**C**) KEGG pathways classification.

Methogenesis, genetic information processing, environmental information processing, cellular activities, organismal systems, human diseases, and drug development are the seven major categories into which the KEGG pathway database groups routes. Each category is divided into several subcategories. Based on this, the targets from the top 20 KEGG terms were classified, and the results are shown in Figure 6C. The majority of genes were classified into the Cancer overview and Neurodegenerative disease subcategories in the Human Diseases category. In the Organismal Systems category, 124 targets were classified into Nervous system, Endocrines system, Sensory system, and immune system subcategories. A total of 31 targets were classified into Lipid metabolism and Energy metabolism subcategories in the Metabolism category; moreover, 107 targets were classified into the Environmental Information Processing category, which includes Signaling molecules and interaction as well as Signal transduction subcategories. The rest of the targets fell into Cell growth and death, and Cellular community-eukaryotes subcategories in the Cellular Processes category. In addition, no targets were found to be classified into the Genetic Information Processing category.

### 2.6. Compound–Target–Pathway Network Construction and Analysis

To further explore the therapeutic mechanism of *Astragalus membranaceus* in treating glioma, the compound–target–pathway network was constructed using Cytoscape 3.6.1 software. Figure 5A shows the network consisted of 352 nodes and 1117 edges (including 330 intersecting targets, 12 molecules and the top 10 KEGG as well as Biological Process pathways). The compounds were ranked according to degree value, from high to low: MOL000131 (degree = 81), MOL000239 (degree = 73), MOL000371 (degree = 52), MOL000378 (degree=50), MOL000380 (degree = 27), MOL000392 (degree = 14), MOL000438 (degree = 7), MOL000432 (degree = 7), MOL000211 (degree = 6), MOL000033 (degree = 3), MOL000296 (degree = 2). The KEGG and Biological Process pathways were ranked based on degree value, from high to low: protein phosphorylation (degree = 108), positive regulation of phosphorylation (degree = 82), cellular response to nitrogen compound cellular (degree = 80), response to organonitrogen compound (degree = 79), peptidyl-amino acid modification (degree = 79), response to hormone (degree = 78), pathways in cancer (degree = 77), regulation of kinase activity (degree = 74), positive regulation of protein phosphorylation (degree = 73), positive regulation of transferase activity (degree = 65).

The compound–core targets network, which has 34 nodes and 85 edges, illustrates the potential interaction mechanism between core targets and compounds (Figure 5C). The compounds were ranked according to degree value, from high to low: MOL000387 (degree = 13), MOL000371 (degree = 10), MOL000438 (degree = 10), MOL000442 (degree = 9), MOL000380 (degree = 8), MOL000392 (degree = 6), MOL000131 (degree = 5), MOL000239 (degree = 5), MOL000378 (degree = 5), MOL000398 (degree = 5), MOL000432 (degree = 5), MOL000033 (degree = 3), MOL000211 (degree = 11). The core targets were ranked according to degree value, the top four core targets are ESR1 (degree = 12), MAPK1 (degree = 6), PIK3CA (degree = 6) and PIK3CB (degree = 6). The degree values of the four central targets PIK3R1, PIK3CB, SRC and PIK3CA are 2, 6, 5, and 6, respectively.

**Figure 7:**
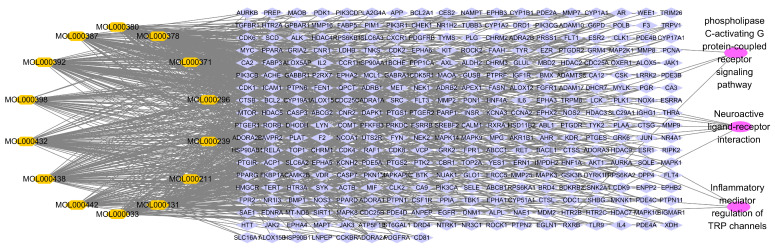
Compound–target–pathway network of CMODE5.

Figure 5B shows that there are seven potential targets for MOL000438, which has no corresponding target in TSMSP database. As indicated in Table 3, the top three enrichment terms of CMODE5 are Neuroactive ligand–receptor interaction, Inflammatory mediator regulation of Transient Receptor Potential (TRP) channels, and phospholipase C-activating G protein-coupled receptor signaling pathway. To further understand the molecular mechanisms, the compound–target–pathway network of CMODE5 was constructed as indicated in Figure 7.

### 2.7. Molecular Docking and Molecular Dynamics Simulations

To verify Vina’s ability to replicate the co-crystallized pose, we performed redocking on four distinct protein targets belonging to various protein families. The findings are displayed in the Supplementary Materials (Figure S1 and Table S5). Assessing the binding mechanisms between herbal substances and disease-associated targets is made possible by the application of molecular docking technologies. By using molecular docking to assess screened active medicines and targets, the results of network pharmacology were verified. Based on the degrees of common targets in the PPI network, core targets identified by CytoHubba and the KEGG results, six targets, which are PIK3R1 (PDB ID: 5GJI), SRC (PDB ID: 7NG7), PIK3CA (PDB ID: 8EXL), JAK2 (PDB ID: 7LL4), AKT1 (PDB ID: 4GV1), and PTK2 (PDB ID: 6YOJ), and 14 compounds were selected for molecular docking. In addition, 14 compounds were docked with three targets, which include PTGS2 (PDB ID: 5F19), ESR1 (PDB ID: 7BAA) and ESR2 (PDB ID: 1QKM), with the highest degree in compound–target–pathway network of CMODE5 to explore the effects of compounds on key proteins in disease pathways. Furthermore, MOL000438, which is a unique component of *Astragalus membranaceus*, was docked with its seven potential targets, which include HDAC7 (PDB ID: 3C10), ROCK1 (PDB ID: 5WNF), HDAC4 (PDB ID: 2VQJ), TBK1 (PDB ID: 4EUU), FLT1 (PDB ID: 4CL7), PDK1 (PDB ID: 2Q8G).

**Figure 8:**
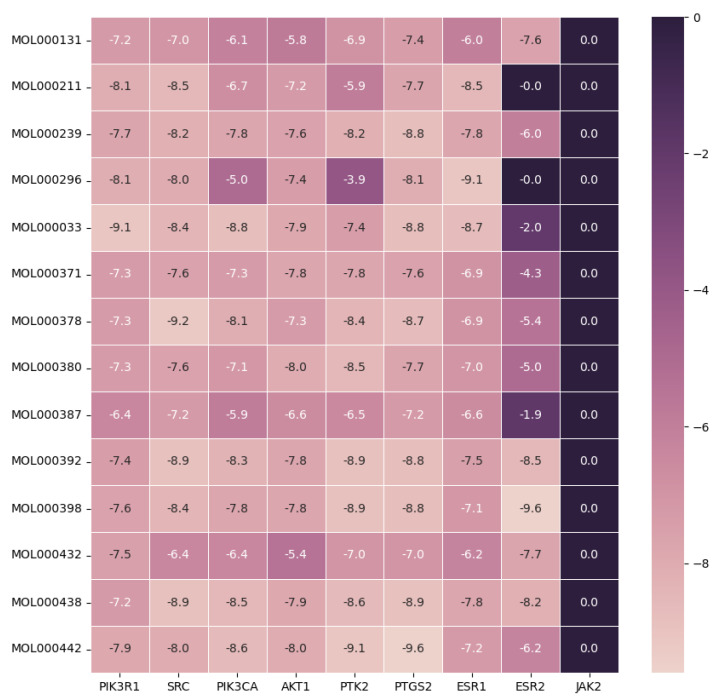
Heatmap of the binding energy (kcal/mol) of key targets and active compounds.

The binding energies (kcal/mol) were obtained from the docking analysis, and the results are shown in Figure 8 and Table 4. A lower score indicates a stronger binding ability. A binding score which is less than −5.0 kcal/mol indicates a moderate affinity, whereas a binding score which is less than −7.0 kcal/mol indicates a high affinity binding. The binding affinity of six complexes (PIK3R1-MOL000033, SRC-MOL000378, PTK2-MOL000442, PTGS2-MOL000442, ESR1-MOL000296 and ESR2- MOL000398) were less than −9.0 kcal/mol, which indicating the stable binding patterns between the active compounds and protein targets.

**Figure 9:**
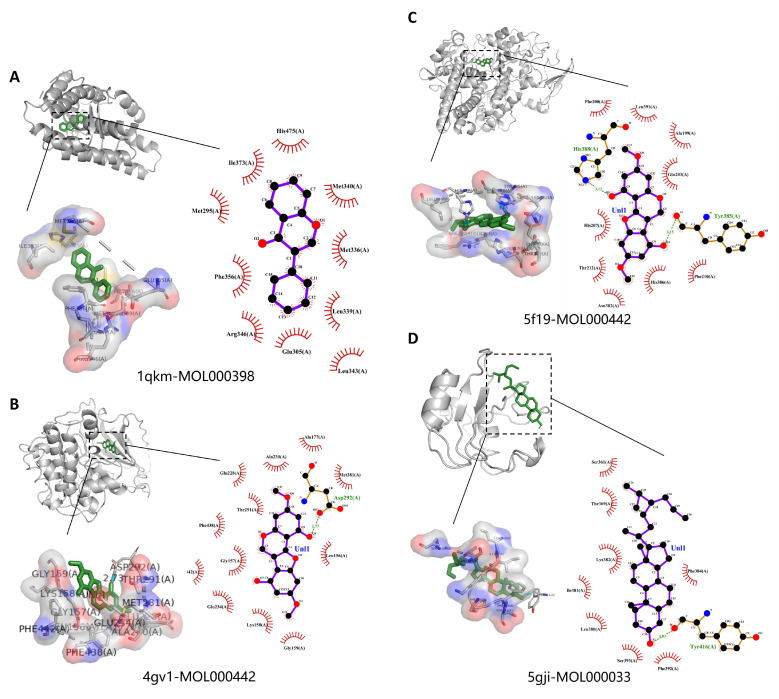

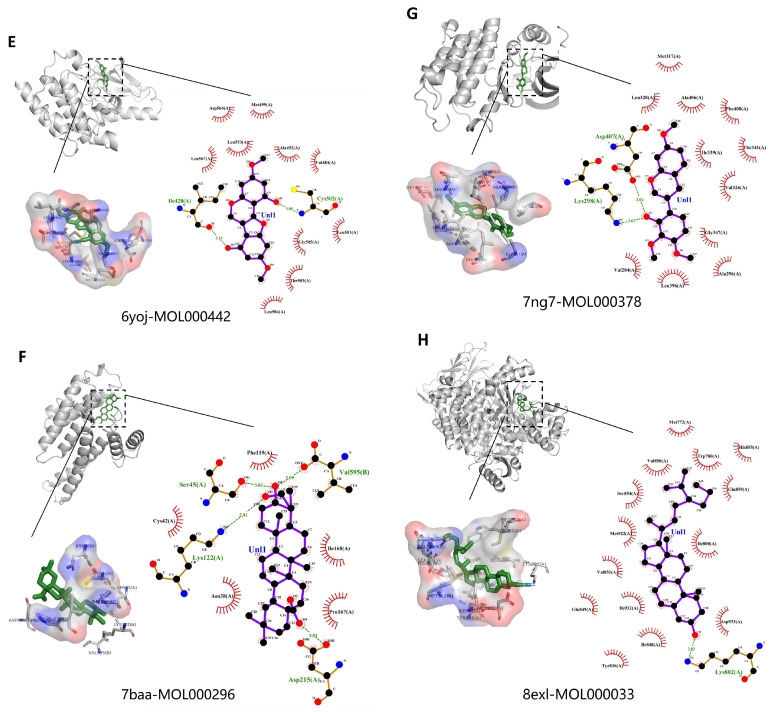
Molecular docking 2D diagram and 3D diagram of key targets and active compounds: (**A**) 1qkm-MOL000398 complex. (**B**) 4gv1-MOL000442 complex. (**C**) 5f19-MOL000442 complex. (**D**) 5gji-MOL000033 complex. (**E**) 6yoj-MOL000442 complex. (**F**) 7baa-MOL000296 complex. (**G**) 7ng7-MOL000378 complex. (**H**) 8exl-MOL000033 complex.

**Figure 10:**
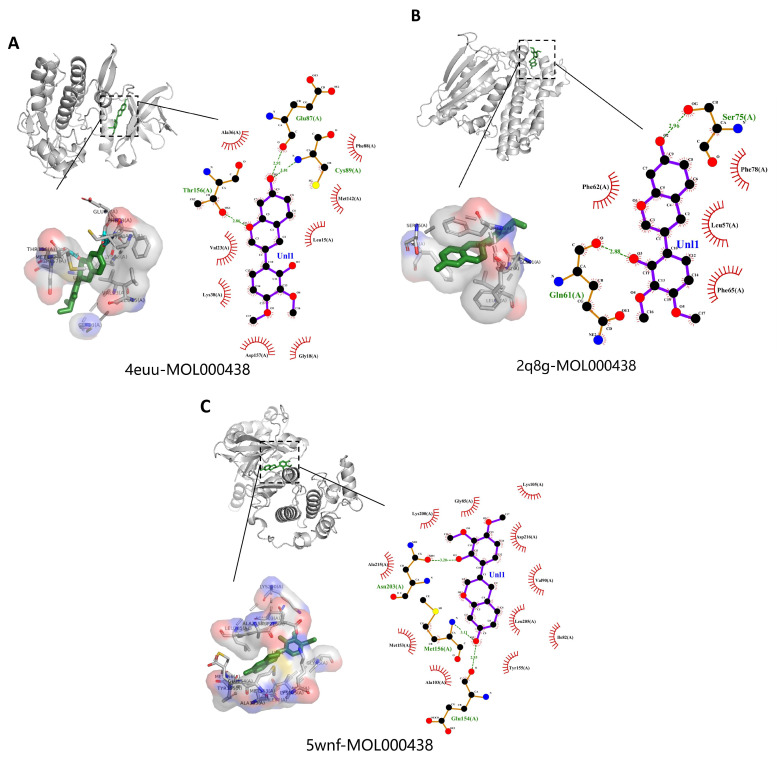
Molecular docking 2D diagram and 3D diagram of MOL000438 and its related targets: (**A**) 4euu-MOL000438 complex. (**B**) 2q8g-MOL000438 complex. (**C**) 5wnf-MOL000438 complex.

PyMOL 2.5.5 and LigPlus were used to visualize the compound–target interaction complexes with the highest free binding energy for each target, along with their binding mechanisms. Figure 9 illustrates the molecular docking 2D diagram and 3D diagram of key targets and active compounds, and Figure 10 shows the molecular docking 2D diagram and 3D diagram of MOL000438 and its related targets. It was discovered that the compounds’ active site residues and the compounds themselves were involved in hydrogen bonding, aromatic stacking (Pi-Sigma, Pi-alkyl, and alkyl interactions), and Van der Waals force.

In order to verify the stability of the ligand–receptor binding, we ran MD simulations on the following complexes: 6yoj-MOL000442; 5wnf-MOL000438; 8exl-MOL000033; 5f19-MOL000442; 4gv1-MOL000442; 2q8g-MOL000438; 5gji-MOL000033; 7baa-MOL000296; 1qkm-MOL000398; 4euu-MOL000438; and 7ng7-MOL000378. The Supplementary Materials (Figures S2–S12 and Tables S6–S25) display the findings of the binding free energy analysis with MM/PBSA and mm/GBSA, as well as the root-mean-square deviation (RMSD), root mean square fluctuation (RMSF), and radius of gyration (Rg). All the complexes are able to bind stably, except for 7baa-MOL000296 complex, which indicates the low probability of 7BAA and MOL000296 interacting, and it also suggests that MOL000296 may have other potential targets. Based on the video in the Supplementary Materials, it appears that the 5f19-MOL000442 complex has multiple low-energy conformations. These findings clarify that, in contrast to Western medicine, the effects of traditional Chinese medicine are obtained gradually through conditioning.

## 3. Discussion

This is the first research looking into the active ingredients in *Astragalus membranaceus* that can cross the blood–brain barrier to treat glioma. Combining data on glioma-related targets from several databases, with network pharmacology and molecular docking techniques allow us to methodically identify the molecular pathways underlying *Astragalus membranaceus*’ anti-glioma actions. Our data demonstrates the correlation between essential chemicals and major protein targets in the occurrence of gliomas, along with the involvement of multiple critical signaling pathways. *Astragalus membranaceus* is a famous widely used Chinese herbal medicine. Its medicinal application was first recorded in Shennong Herbal Classic, which was written in the Han Dynasty [32]. *Astragalus membranaceus* has been primarily utilized in Chinese herbology to treat infections of the gastrointestinal and respiratory tracts, carbuncles, diarrhea, pyrexia, and jaundice [33,34]. Glioma is one of the most lethal types of brain tumor, and the therapies for this dreadful disease were not enough. Several compounds isolated from *Astragalus membranaceus* are approved to have anti-tumor, anti-oxidant, anti-inflammatory, neuroprotective, and other pharmacological activities [35,36]. Even if some research has indicated that *Astragalus membranaceus* and its extracts have anti-glioma properties [22,37,38,39,40,41], the use of molecular docking and computational pharmacological techniques to methodically investigate the active ingredients, possible targets, and pharmacological mechanism of *Astragalus membranaceus* in the treatment of gliomas is still lacking. Furthermore, it is unknown what numerous targets and paths its anti-glioma effects take. In terms of methodically deciphering the molecular basis of Traditional Chinese Medicine and illustrating the possible interactions between multiple targets and herb components, network pharmacology is superior. In order to investigate the possible pharmacological and molecular mechanisms of *Astragalus membranaceus* against glioma, we conducted network pharmacology. A total of 14 active compounds and 343 putative targets linked to glioma were discovered; several targets were found to be impacted by multiple compounds, suggesting that the *Astragalus membranaceus* active chemicals may modulate and interact with multiple glioma-related targets.

According to the results of PPI analysis of the 343 targets, the top ten targets, including SRC1, PIK3R11, HSP90AA11, PIK3CA1, PIK3CB1 PIK3CD1, AKT11. ESR11, MAPK11, and HSP90AB11, may be the key targets of the treatment for glioma. We conducted GO and KEGG enrichment analyses on the 343 chosen targets in order to have a more systematic understanding of the various effects of *Astragalus membranaceus* for treating gliomas. The active chemicals may participate in the MFs, CCs, and BPs to exert their effects, according to the top 10 GO functional categories. The majority of the top 20 BP phrases are connected to inflammation, hormone control, and different forms of phosphorylation. Since many enzymes and receptors are activated or deactivated by phosphorylation and dephosphorylation processes, which are carried out by kinases and phosphatases, protein phosphorylation is an essential regulator of protein and cellular function [42,43]. While phosphorylation-related pathways are closely linked to a number of malignancies, they may also be targets for cancer-fighting medications [42,44,45,46]. The screened targets in phosphorylation pathways may be potent anti-glioma drug targets and represent a promising use for glioma therapy. The terms of MF are closely associated with various kinase activities as well as G protein-coupled amine receptor activity. Combined with all the results of GO enrichment analysis, *Astragalus membranaceus* may play an anti-glioma role in regulating different kinase activities and G protein-coupled receptor activities. The results of the KEGG pathway enrichment analysis indicate that 343 targets are significantly enriched in 215 signaling pathways, which indicates that *Astragalus membranaceus* can target multiple pathways simultaneously. Most of the enriched KEGG terms are categorized under the categories of Cancer, Neurodegenerative disease, and Signaling molecules and interaction. Among these 215 signaling pathways, the hsa04151: PI3K-Akt signaling pathway is responsible for growth, cell proliferation, and metabolism [47], which was reported by other researchers who found that through decreased AKT and FAK activation, PIK3CA knockdown significantly decreases cell survival, migration, and invasion of GBM cell [48]. PIK3CA, which is one of the central targets identified through PPI analysis in our work, presents high binding affinities with several compounds from *Astragalus membranaceus*, namely (3S,8S,9S,10R,13R,14S,17R)-10,13-dimethyl-17-[(2R,5S)-5-propan-2-yloctan-2-yl]-2,3,4,7,8,9,11,12,14,15,16,17-dodecahydro-1H-cyclopenta[a]phenanthren-3-ol(MOL000033), 1,7-Dihydroxy-3,9-dimethoxy pterocarpene(MOL000442), (3R)-3-(2-hydroxy-3,4- dimethoxyphenyl)chroman-7-ol (MOL000438), formononetin (MOL000392), and Bifendate (MOL000387). This finding indicates that these compounds could be the potential drugs targeting the important glioma-related target PIK3CA.

(3R)-3-(2-hydroxy-3,4-dimethoxyphenyl)chroman-7-ol(MOL000438) [49,50], which is unique to *Astragalus membranaceus*, has no reported targets. In this research, we studied the molecular mechanism of (3R)-3-(2-hydroxy-3,4-dimethoxyphenyl)chroman-7-o through molecular docking, and found that this compound may target multiple glioma-related targets, such as SRC (binding affinity = −8.9 kcal/mol), PTGS2 (binding affinity = −8.9 kcal/mol), and PTK2 (binding affinity = −8.6 kcal/mol).

Moreover, to further explore and understand the potential function and interaction of the screened targets, the enrichment analysis of MCODE clusters discovered that several MCODE clusters are related to protein phosphorylation, which has been discussed in several studies [51,52,53,54]. Additionally, the PI3K-Akt signaling pathway, as well as pathways in cancer, are significantly enriched in MCODE1, and this result has been well explored in former studies [55,56,57]. In addition, phospholipase C-activating G protein-coupled receptor signaling pathway, which the signal is transmitted via the activation of phospholipase C (PLC) and a subsequent increase in the intracellular concentration of inositol trisphosphate (IP3) and diacylglycerol (DAG), was enriched in MCODE5 cluster. A previous study has demonstrated that G-protein-coupled receptor GPR17 inhibits glioma development by increasing polycomb repressive complex 1-mediated ROS production. Additionally, another interesting finding is that inflammatory mediator regulation of Transient Receptor Potential (TRP) channels was also significantly enriched in CMODE5. A recent study discovered that upregulation of HTR2A, which is a target in inflammatory mediator regulation of Transient Receptor Potential (TRP) channels, and COMT was significantly positively correlated with glioma carcinogenesis. The prognosis for glioma patient survival may be further impacted by the expression of HTR2A and COMT, which could further impact the effectiveness of immunotherapy. Additionally, HTR2A expression may positively correlate with tumor heterogeneity and have an impact on patient prognosis and survival [58]. From the above results, it can be seen that the active compounds in *Astragalus membranaceus* can regulate multiple pathways simultaneously to affect glioma.

Finally, molecular docking was performed to evaluate nine key target proteins (PIK3R1, SRC, PIK3CA, JAK2, AKT1, PTK2, PTGS2, ESR1, and ESR2) and 14 active compounds, including EIC, Mairin, Jaranol, Hederagenin, 2,14,15,16,17- dodecahydro-1H-cyclopenta[a]phenanthren-3-ol, 3,9-di-O-methylnissolin, isoflavanone, Bifendate, Formononetin, 7-O-methylisomucronulatol, (6aR,11aR)-9,10-dimethoxy-6a,11a-dihydro-6H-benzofurano[3,2-c]chromen-3-ol, linolenic acid, (3R)-3-(2-hydroxy-3,4- dimethoxyphenyl)chroman-7-ol, 1,7-Dihydroxy-3,9-dimethoxy pterocarpene, (3S,8S,9S,10R,13R,14S,17R)-10,13-dimethyl-17-[(2R,5S)-5-propan-2-yloctan-2-yl]-2,3,4,7,8,9,11. Two complexes, the ESR2-isoflavanone complex and the PTGS2-1,7-Dihydroxy-3,9-dimethoxy pterocarpene complex, had the lowest binding affinities (−9.6 kcal/mol), suggesting that they could be the main active ingredients and targets of *Astragalus membranaceus* in the treatment of glioma.

While *Astragalus membranaceus* has been examined during the past few years for its therapeutic benefit in the treatment of gliomas, not enough has been done to explore its mechanism. This study is the first to systematically analyze the molecular mechanism of *Astragalus membranaceus* for the treatment of glioma, using the network pharmacology and molecular docking approach. Furthermore, there is still a pressing need for innovative glioma treatment approaches. In this work, we offer a number of viable targets for glioma treatment, which may aid in the creation of fresh therapeutic approaches. It is important to note that this study has certain limitations. Initially, the bioactive component of *Astragalus membranaceus* and targets associated with gliomas were retrieved from the databases; hence, the dependability and precision of the analysis and forecast rely on the quality of the data. Secondly, additional in vivo and in vitro research is required to validate the results.

## 4. Materials and Methods

### 4.1. Screening of Active Compounds and Potential Targets of Astragalus membranaceus

Traditional Chinese Medicine Systems Pharmacology (TCMSP, https://tcmsp-e.com/tcmsp.php, accessed on 1 August 2023) is a systems pharmacology platform for Chinese herbal medicines, provides interactive data related to the relationships between drugs, targets, and diseases [59]. This platform also provides information on the pharmacokinetic effects of natural compounds, including drug-likeness, oral bioavailability, intestinal epithelial permeability, aqueous solubility, and blood–brain barrier permeability. In this study, TCMSP was utilized to search for active compounds for *Astragalus membranaceus* and active compounds were screened under the conditions of drug-likeness (DL) ≥ 0.18, which is determined upon the fact that the average DL index in the Drugbank [60] is 0.18, Oral Bioavailability (OB) ≥ 30%, which was calculated using OBioavail [61] from previous research, and BBB ≥ −0.3, which indicates that compounds that meet this condition can penetrate the blood–brain barrier, which is a very important parameter in the treatment of glioma [59].

The related targets of the active compounds obtained in the above steps were collected in TCMSP database, and the target protein names were converted to the official gene symbols using Perl (http://www.perl.org/) and UniProt (http://www.UniProt.org/, accessed on 1 August 2023) databases [62]. Unmatched gene symbols were supplemented by searching from Drugbank database and literature review. Additionally, the SMILES formats of the active compounds were obtained from PubChem database (https://pubchem.ncbi.nlm.nih.gov/, accessed on 1 August 2023) [63], then were imported into the SwissTargetPrediction platform (http://www.swisstargetprediction.ch/, accessed on 1 August 2023) [64,65] in order to search for the potential targets with the species set as “Homo sapiens” and the probability set larger than 0.

### 4.2. Construction of Glioma-Related Targets Dataset

Targets related to glioma were retrieved by using the keyword “glioma” in the GeneCards database (https://www.genecards.org/, accessed on 12 August 2023) [66]. All targets contained in UMLS CUI: C0541798 were collected from DisGeNET database (https://www.disgenet.org, accessed on 12 August 2023) [67]. Additionally, all the targets related to glioma were downloaded from Brainbase database (https://ngdc.cncb.ac.cn/brainbase/, accessed on 13 August 2023) [68,69], which is based on manual curation of 2768 published articles about brain disease along with information retrieval from several public databases. A glioma-related targets dataset was constructed by combining all the targets from these three databases and removing duplicates.

### 4.3. Common Targets of Astragalus membranaceus in Treating Glioma

Glioma-related targets and potential targets of *Astragalus membranaceus* were imported into VENN DIAGRAMS (http://bioinformatics.psb.ugent.be/webtools/Venn/, accessed on 29 August 2023) to acquire intersection targets.

### 4.4. Protein–Protein Interaction (PPI) Network Construction and MCODE Modules Analysis

Common targets of glioma-related targets and *Astragalus membranaceus* targets were imported into the STRING database (http://string-db.org, accessed on 29 August 2023) [70], which is an open-source database that builds a Protein–Protein Interaction PPI network by integrating a variety of information on interactions between proteins from other internet sources. “Homo Sapiens” was the definition of the organism, “highest confidence” (>0.9) was the minimal interaction threshold, and the rest were set as the default. Then, the PPI result was imported into Cytoscape 3.6.1 software [71] to construct a PPI network, and the isolated nodes were removed. Meanwhile, the cytohubba tool in Cytoscape was used to calculate the top 10 targets by Degree, Maximum Neighborhood Component (MNC), Maximal Clique Centrality (MCC), and Closeness algorithms, and the intersecting targets were defined as the central targets [72].

The Metascape platform (http://metascape.org, accessed on 31 August 2023) [73] was used to analysis biological process of interaction of the common targets and to obtain the top functional modules using molecular complex detection (MCODE), which uses a vertex-weighting scheme to discover local, high-density areas in the PPI graph [29].

### 4.5. Gene Ontology (GO) and Kyoto Encyclopedia of Genes and Genomes (KEGG) Enrichment Analysis

In order to obtain a more systematic and comprehensive understanding of the mechanisms behind the targets, Gene Ontology (GO), which includes biological processes, cellular components, and molecular functions [74], as well as the Kyoto Encyclopedia of Genes and Genomes (KEGG) [75]. Enrichment analysis of the obtained intersection targets were performed using Metascape (http://Metascape.org, accessed on 31 August 2023). The species was set to “Homo Sapiens”, the *p*-value was set to “<0.01”, the smallest count was set to “3”, and the enrichment factor was set to “>1.5”. The heatmap was plotted by https://www.bioinformatics.com.cn, accessed on 31 August 2023), an online platform for data analysis and visualization.

### 4.6. Construction of a Compound–Target–Pathway Network

A compound–target–pathway was constructed and examined using Cytoscape 3.6.1 to illustrate and clarify the intricate relationships between active compounds, pathways, and targets in order to better investigate the regulatory mechanism of the active compounds against glioma. The top 20 enriched KEGG pathways, active compounds in *Astragalus membranaceus* that satisfy screening criteria, and glioma-related core targets were utilized in this study to build the compound–target–pathway interaction network.

### 4.7. Molecular Docking and Molecular Dynamics Simulations

Molecular docking was performed using the 14 compounds from *Astragalus membranaceus* and the top 8 target proteins selected from PPI and and MCODE analysis results. The 3D structures of the target protein (PDB format) were downloaded from the PDB database (https://www.rcsb.org, accessed on 3 September 2023) [76]. PyMOL 2.5.5 was used to extract the target protein’s water molecules, original ligands, and peptide. After that, AutoDock Tools 1.5.7 was used to import the target proteins and process them for hydrogenation, charge calculation, and non-polar hydrogen combination. The PDBQT format was then used to export the treated protein structures. For PIK3R1, SRC, PIK3CA, AKT1, PTK2, PTGS2, ESR1, ESR2, JAK2, HDAC7, ROCK1, HDAC4, TBK1 and PDK1, the active pocket’s locations were built depending on the original ligands’ or original peptides’ positions in the 3D structures, and the size of the grid Box and the X, Y and Z centers were adjusted based on the original ligands or peptides of the receptor. For FLT1, the size of gird Box was set to 15 15 15, and the coordinates of center were set to center_x = −18.367, center_y = 13.527 and center_z = 29.601. While the TCMSP Database and the PubChem Database provided the compounds’ 3D structures (in Mol2 format). Employing AutoDock Tools 1.5.7, the compound rotation bond was configured and additionally preserved in pdbqt format.

The AutoDock Vina [77,78] CMD command was used for docking and searching for the optimal conformation of PIK3R1, SRC, PIK3CA, AKT1, PTK2, PTGS2, ESR1, ESR2, JAK2, HDAC7, ROCK1, HDAC4, TBK1, and PDK1. For FLT1, molecular docking was performed using qvina-w [47], which utilizes the powerful scoring function of AutoDock Vina and is more suitable for blind docking. The exhaustiveness was set to 24, and the other parameters were default.

Molecular dynamics simulations and binding free energy calculations were performed on the best docked coordinates of each complex using GROMACS (version 2023.1) [79].

The results were carefully analyzed and visualized by PyMOL 2.5.5 [80], LigPlus v.2.2 tools and Python seaborn v0.13.

## 5. Conclusions

This study elaborated the multi-component, multi-target, and multi-pathway properties of the bioactive component of *Astragalus membranaceus* in treating glioblastoma utilizing network pharmacology and molecular docking. We discovered two potent compounds, isoflavanone and 1,7-Dihydroxy-3,9-dimethoxy pterocarpene, that may effectively connect with the respective targets ESR2 and PTGS2, as well as four main targets, namely PIK3R1, PIK3CB, SRC, and PIK3CA. These findings provide a research foundation for further investigation of *Astragalus membranaceus* in anti-glioma.

## Figures and Tables

**Table 1:** Active compounds and their targets from TCMSP database.

Mol ID	Molecule Name	OB (%)	BBB	DL	Targets Number
MOL000131	EIC	41.9	0.9	0.14	15
MOL000211	Mairin	55.38	0.22	0.78	1
MOL000239	Jaranol	50.83	−0.22	0.29	13
MOL000296	hederagenin	36.91	0.96	0.75	24
MOL000033	(3S,8S,9S,10R,13R,14S,17R)-10,13-dimethyl-17-[(2R,5S)-5-propan-2-yloctan-2-yl]-2,3,4,7,8,9,11,12,14,15,16,17-dodecahydro-1H-cyclopenta[a]phenanthren-3-ol	36.23	1.09	0.78	1
MOL000371	3,9-di-O-methylnissolin	53.74	0.63	0.48	23
MOL000378	7-O-methylisomucronulatol	74.69	0.84	0.3	45
MOL000380	(6aR,11aR)-9,10-dimethoxy-6a,11a-dihydro-6H-benzofurano[3,2-c]chromen-3-ol	64.26	0.55	0.42	22
MOL000387	Bifendate	31.1	−0.06	0.67	7
MOL000392	formononetin	69.67	0.02	0.21	39
MOL000398	isoflavanone	109.99	0.17	0.3	0
MOL000432	linolenic acid	45.01	0.84	0.15	40
MOL000438	(3R)-3-(2-hydroxy-3,4-dimethoxyphenyl)chroman-7-ol	67.67	0.34	0.26	0
MOL000442	1,7-Dihydroxy-3,9-dimethoxy pterocarpene	39.05	−0.04	0.48	4

**Table 2:** Common genes calculated by MCC, MNC, Degree, and Closeness.

Target	MCC Score	MNC Score	Degree Score	Closeness Score
PIK3R1	5,340,457	36	37	122.8464286
PIK3CB	5,385,600	35	35	119.0325397
SRC	4,523,488	35	39	128.1369048
PIK3CA	5,385,744	35	35	121.8464286

**Table 3:** Results of top three functional modules of MCODE1, MCODE2, MCODE3, MCODE4, MCODE5, MCODE6, and MCODE7.

MCODE	GO	Description	Log10 (P)
MCODE_1	hsa05200	Pathways in cancer	−80.9
MCODE_1	GO:0006468	protein phosphorylation	−79.3
MCODE_1	hsa04151	PI3K-Akt signaling pathway	−65.4
MCODE_2	GO:0006468	protein phosphorylation	−14.3
MCODE_2	GO:0048511	rhythmic process	−12.9
MCODE_2	GO:0042327	positive regulation of phosphorylation	−11.2
MCODE_3	GO:0006468	protein phosphorylation	−15.3
MCODE_3	GO:0062197	cellular response to chemical stress	−9.1
MCODE_3	GO:0046777	protein autophosphorylation	−9.1
MCODE_4	GO:1901361	organic cyclic compound catabolic process	−10.6
MCODE_4	GO:0001505	regulation of neurotransmitter levels	−9.7
MCODE_4	GO:0042424	catecholamine catabolic process	−9.6
MCODE_5	hsa04080	Neuroactive ligand–receptor interaction	−8.4
MCODE_5	hsa04750	Inflammatory mediator regulation of Transient Receptor Potential (TRP) channels	−6.2
MCODE_5	GO:0007200	phospholipase C-activating G protein-coupled receptor signaling pathway	−6.1
MCODE_6	hsa04080	Neuroactive ligand–receptor interaction	−12.9
MCODE_6	GO:0007210	serotonin receptor signaling pathway	−11.5
MCODE_6	GO:1903351	cellular response to dopamine	−9.6
MCODE_7	hsa04080	Neuroactive ligand–receptor interaction	−6.1
MCODE_7	GO:0098660	inorganic ion transmembrane transport	−4.9
MCODE_7	GO:0042391	regulation of membrane potential	−4

**Table 4:** Table of binding energy (kcal/mol) of MOL000438 and its related targets.

Compound	Targets					
MOL000438	HDAC7	ROCK1	HDAC4	TBK1	FLT1	PDK1
	−8.2	−8.5	−7.2	−8.8	−6.3	−8.6

## Data Availability

The original contributions presented in the study are included in the Supplementary Materials. Further inquiries can be directed to the corresponding authors.

## Supplementary Materials

The following supporting information can be downloaded at: https://www.mdpi.com/article/10.3390/ijms242216306/s1.
