# Supplementary material for: A Network Pharmacology Prediction and Molecular Docking-Based Strategy to Explore the Potential Pharmacological Mechanism of Astragalus membranaceus for Glioma
Source: Int J Mol Sci. 2023 Nov 14;24(22):16306. doi: 10.3390/ijms242216306 (PMC10671347; doi:10.3390/ijms242216306)
Supplement: Supplementary file 1 [file ijms-24-16306-s001.zip › Figure S2. - Figure S12. Results of molecular dynamics simulations_20231029083815.pdf]

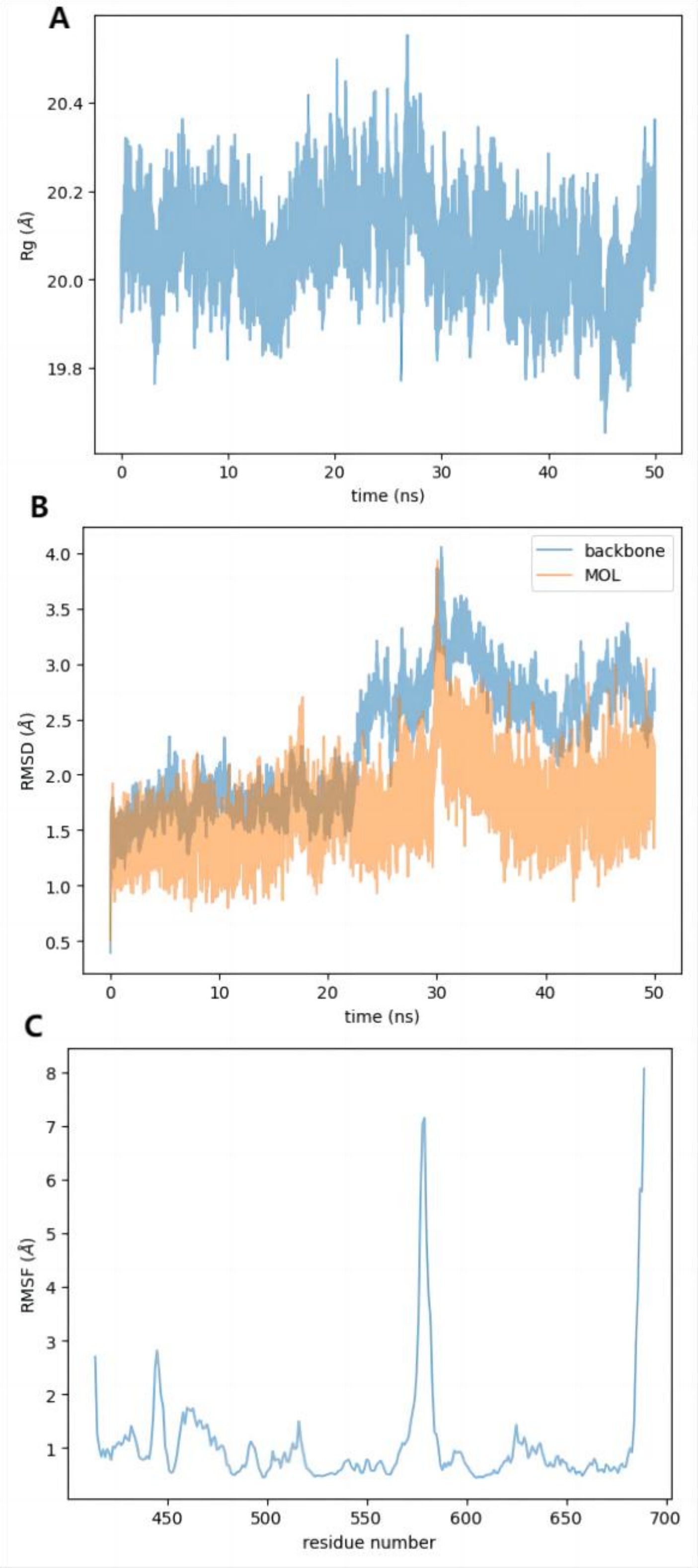

Figure S2. Results of molecular dynamics simulation of 6yoj-MOL000442 complex. (A) Rg plot of 6yoj-MOL000442 complex. (B) RMSD plot of 6yoj-MOL000442 complex. (C) RMSF plot of 6yoj-MOL000442 complex.

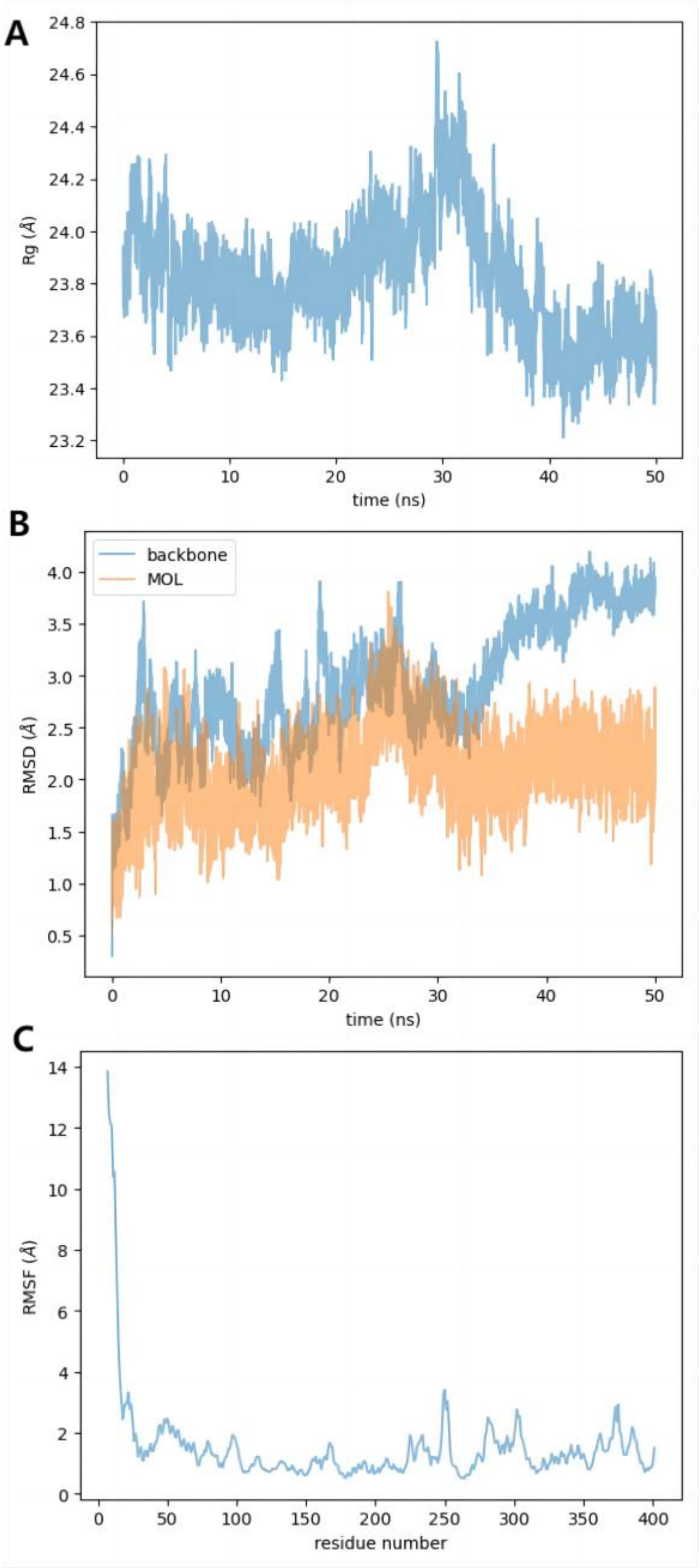

Figure S3. Results of molecular dynamics simulation of 5wnf-MOL000438 complex. (A) Rg plot of 5wnf-MOL000438 complex. (B) RMSD plot of 5wnf-MOL000438 complex. (C) RMSF plot of 5wnf-MOL000438 complex.

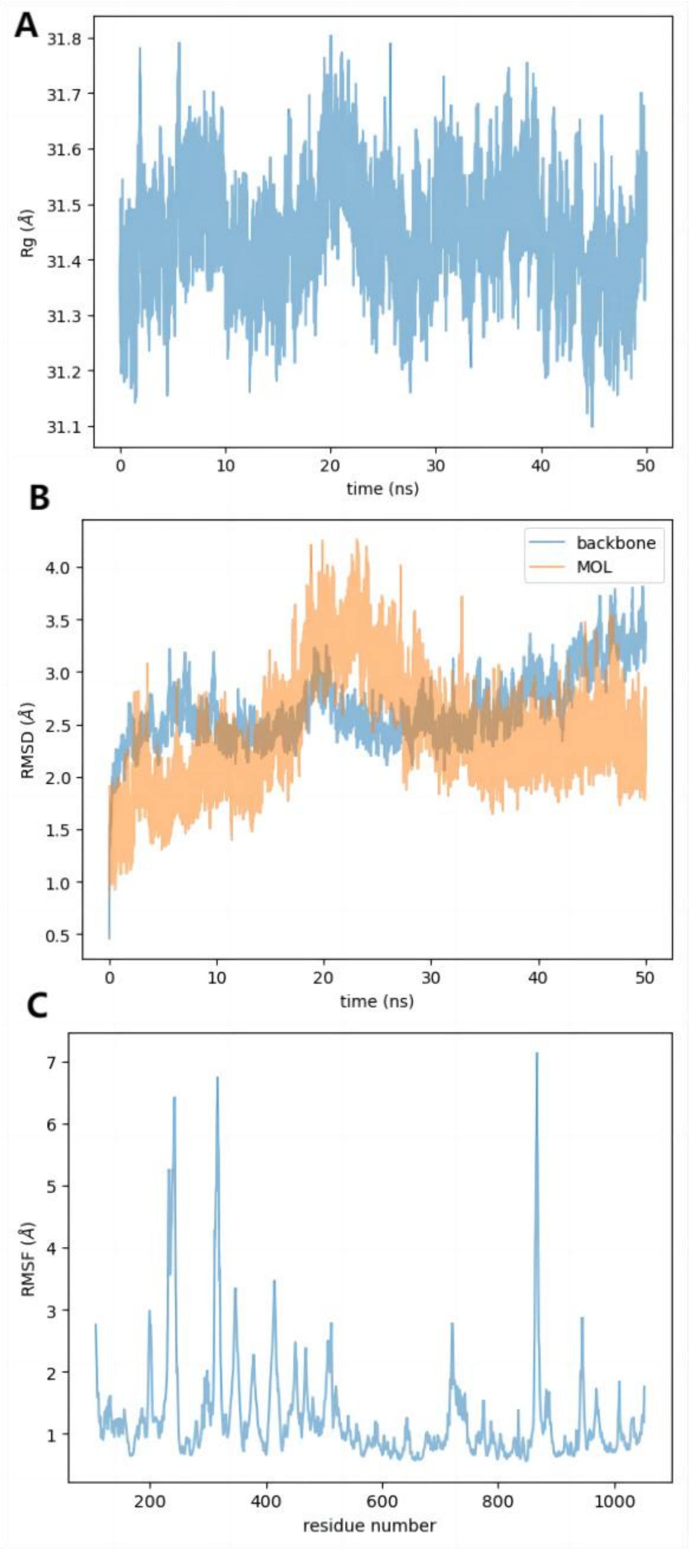

Figure S4. Results of molecular dynamics simulation of 8exl-MOL000033 complex. (A) Rg plot of 8exl-MOL000033 complex. (B) RMSD plot of 8exl-MOL000033 complex. (C) RMSF plot of 8exl-MOL000033 complex.

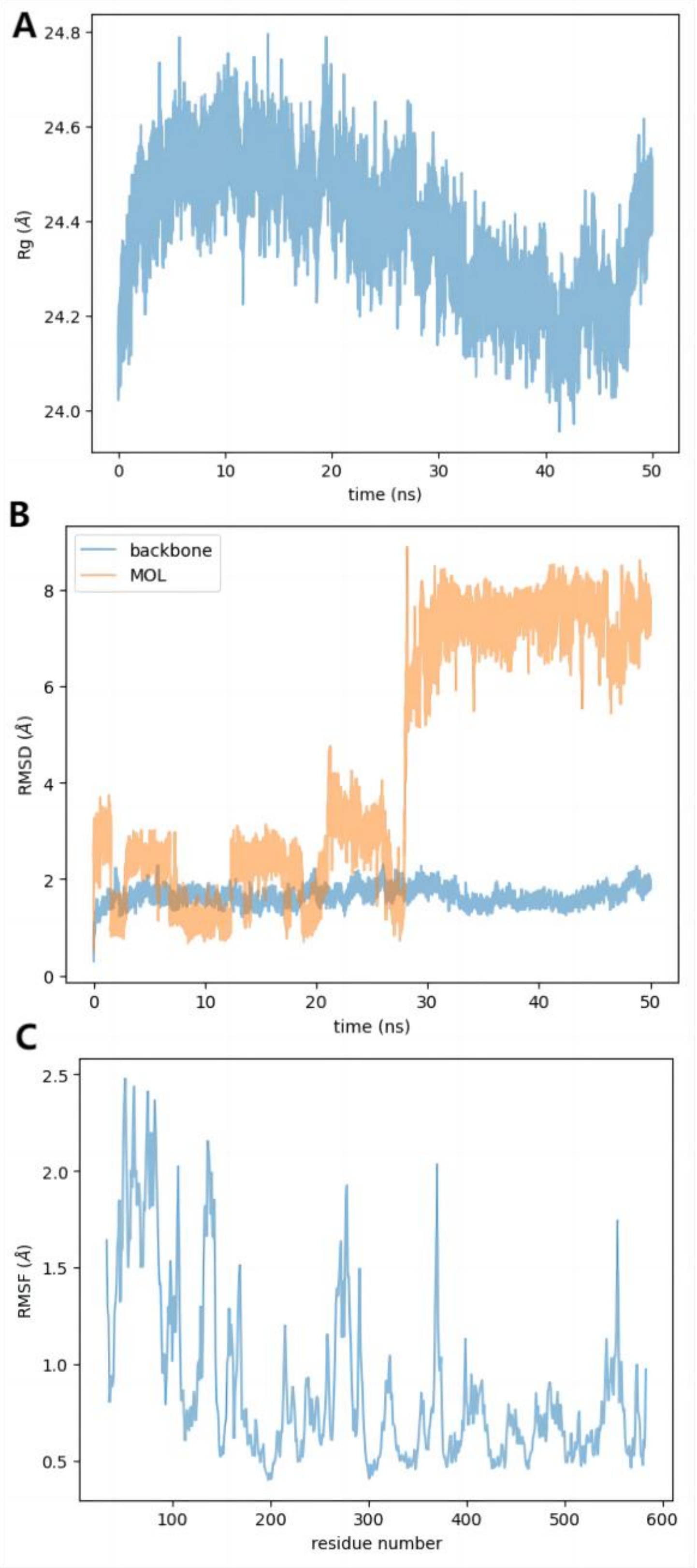

Figure S5. Results of molecular dynamics simulation of 5f19-MOL000442 complex. (A) Rg plot of 5f19-MOL000442 complex. (B) RMSD plot of 5f19-MOL000442 complex. (C) RMSF plot of 5f19-MOL000442 complex.

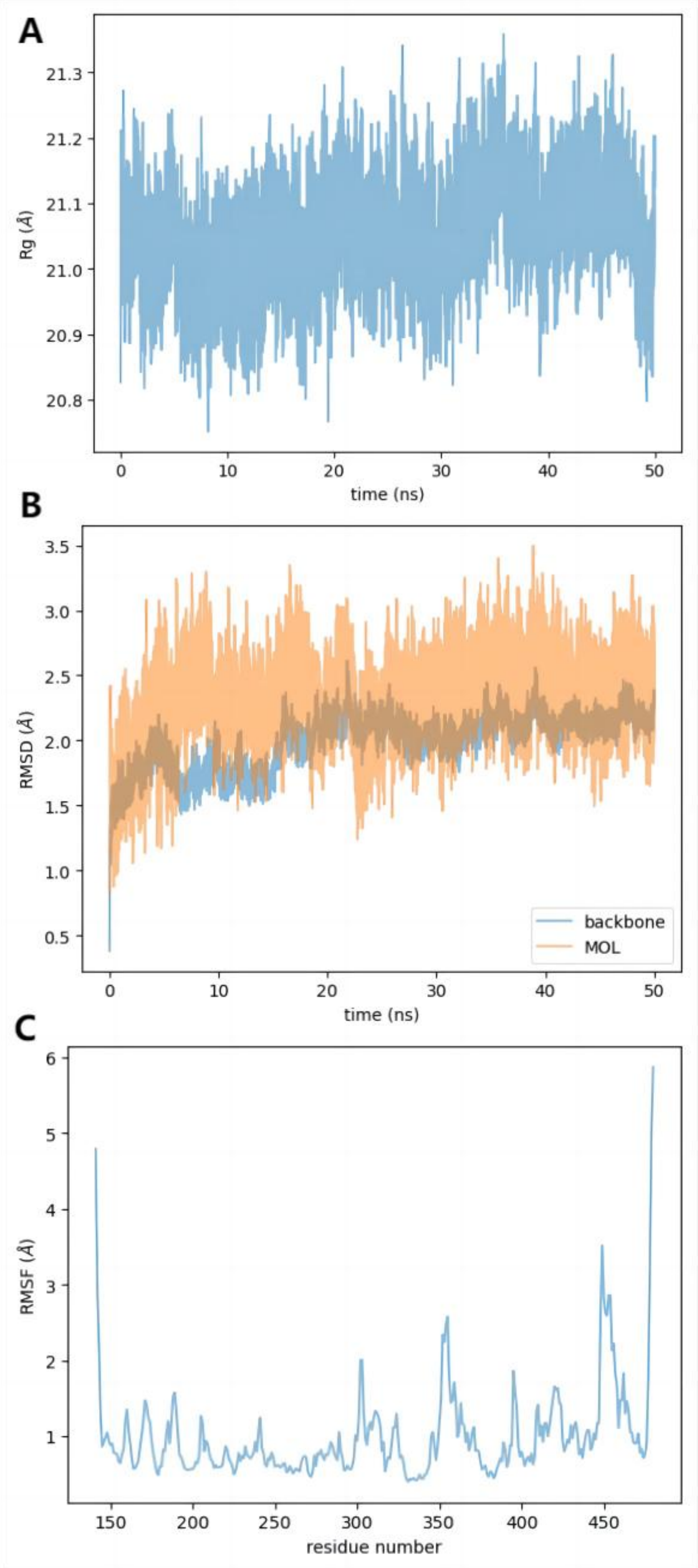

Figure S6. Results of molecular dynamics simulation of 4gv1-MOL000442 complex. (A) Rg plot of4gv1-MOL000442 complex. (B) RMSD plot of 4gv1-MOL000442 complex. (C) RMSF plot of 4gv1-MOL000442 complex.

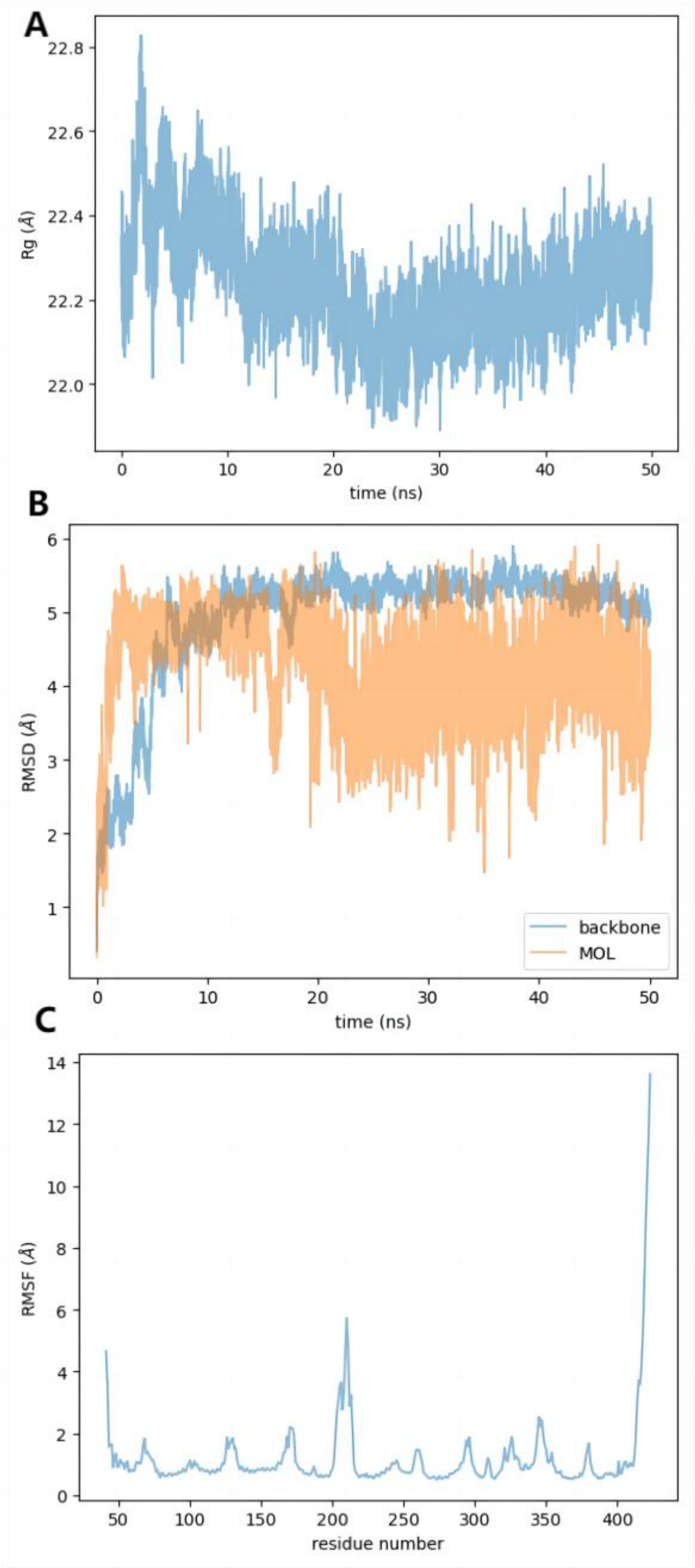

Figure S7. Results of molecular dynamics simulation of 2q8g-MOL000438 complex. (A) Rg plot of2q8g-MOL000438 complex. (B) RMSD plot of 2q8g-MOL000438 complex. (C) RMSF plot of 2q8g-MOL000438 complex.

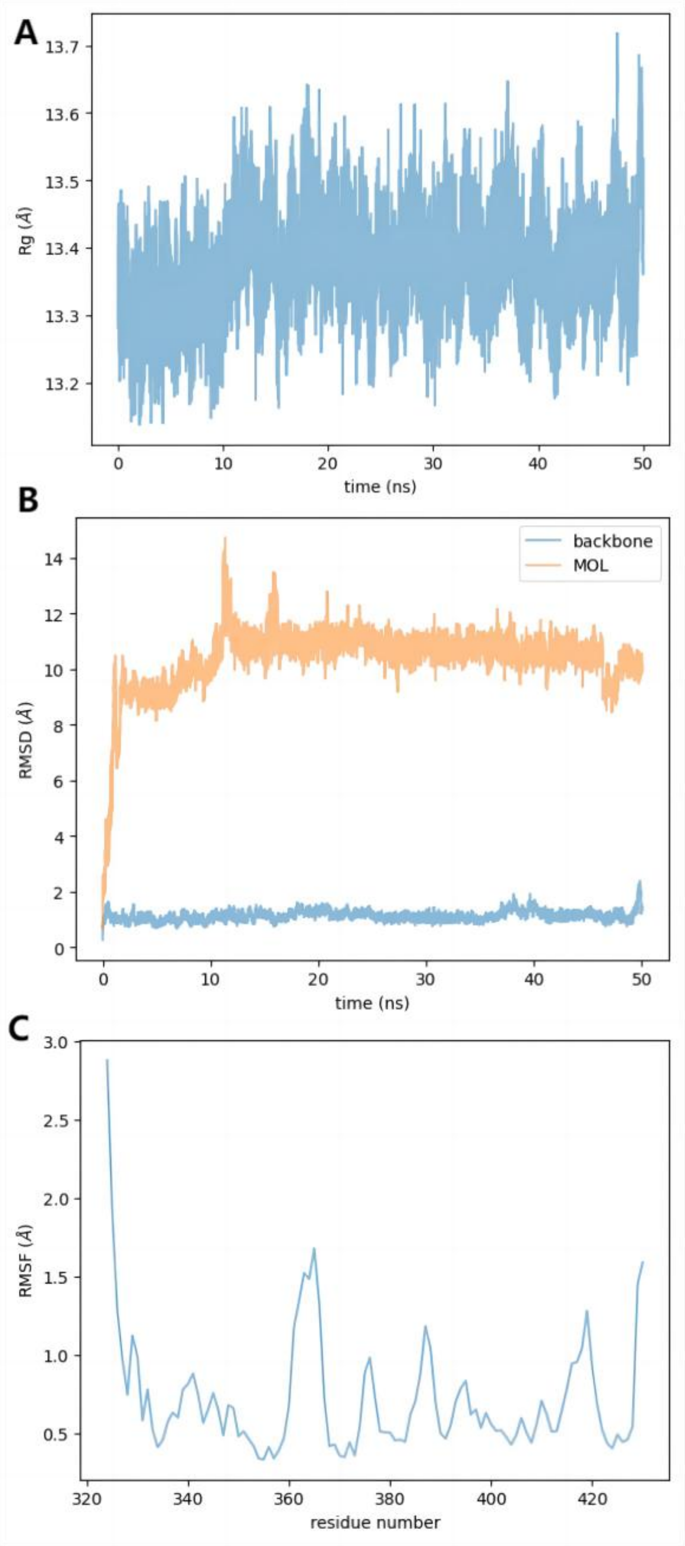

Figure S8. Results of molecular dynamics simulation of 5gji-MOL000033 complex. (A) Rg plot of 5gji-MOL000033 complex. (B) RMSD plot of 5gji-MOL000033 complex. (C) RMSF plot of 5gji-MOL000033 complex.

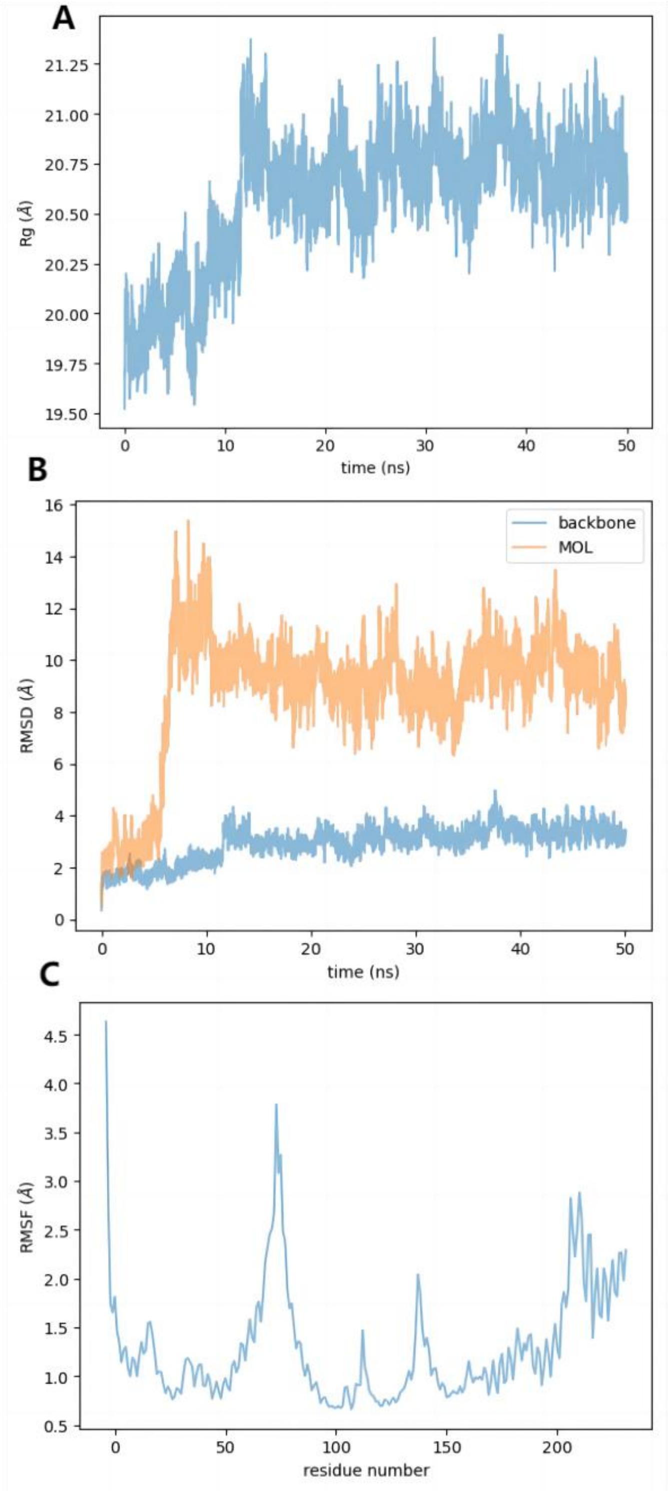

Figure S9. Results of molecular dynamics simulation of 7baa-MOL000296 complex. (A) Rg plot of 7baa-MOL000296 complex. (B) RMSD plot of 7baa-MOL000296 complex. (C) RMSF plot of 7baa-MOL000296 complex.

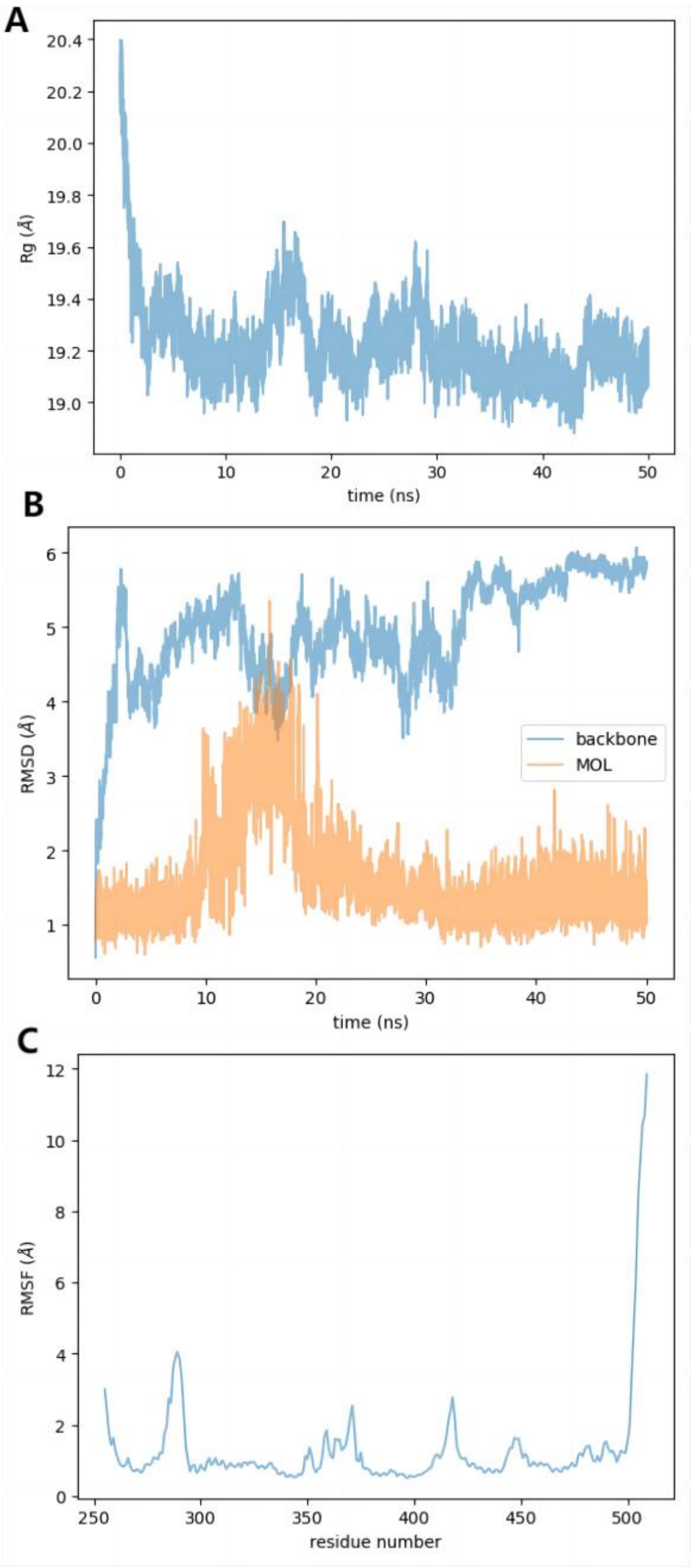

Figure S10. Results of molecular dynamics simulation of 1qkm-MOL000398 complex. (A) Rg plot of 1qkm-MOL000398 complex. (B) RMSD plot of 1qkm-MOL000398 complex. (C) RMSF plot of 1qkm-MOL000398 complex.

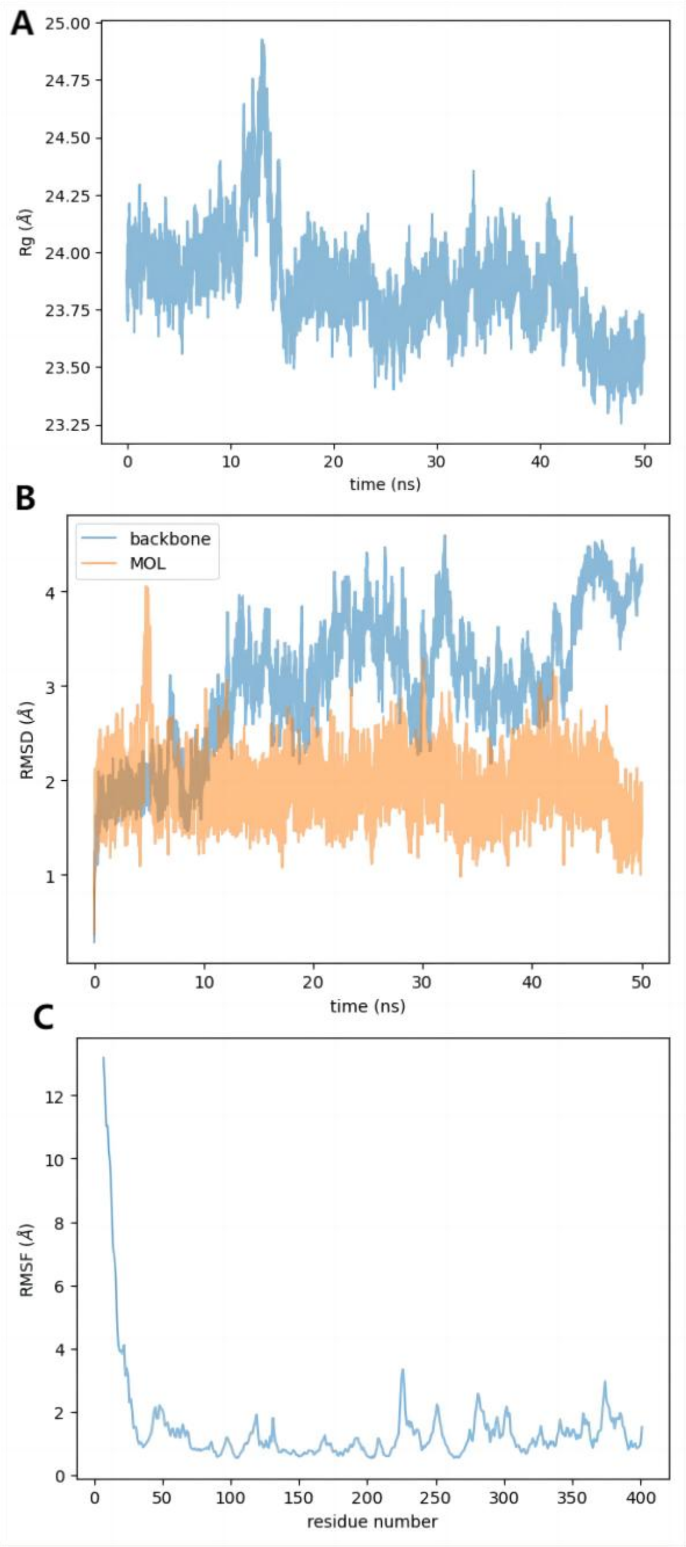

Figure S11. Results of molecular dynamics simulation of 4euu-MOL000438 complex. (A) Rg plot of 4euu-MOL000438 complex. (B) RMSD plot of 4euu-MOL000438 complex. (C) RMSF plot of 4euu-MOL000438 complex.

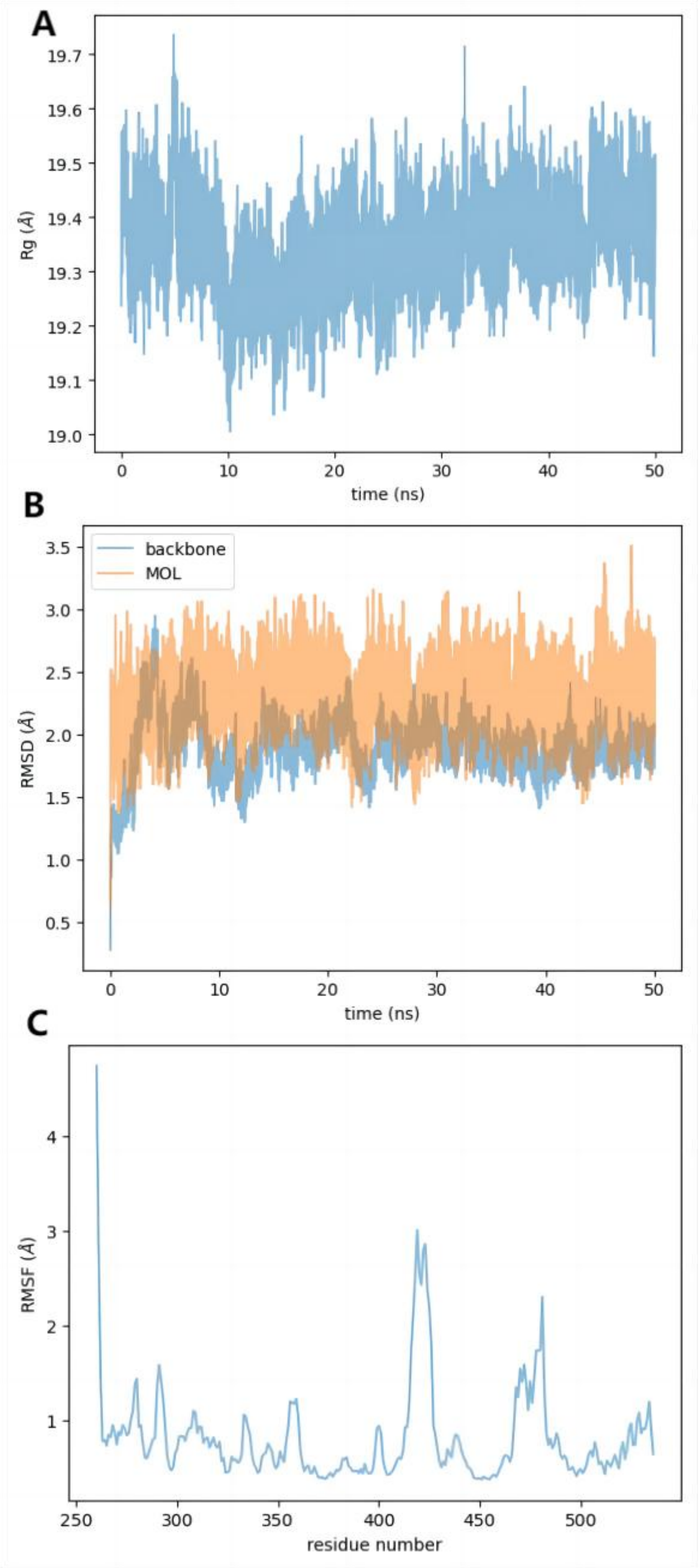

Figure S12. Results of molecular dynamics simulation of 7ng7-MOL000378 complex. (A) Rg plot of 7ng7-MOL000378 complex. (B) RMSD plot of 7ng7-MOL000378 complex. (C) RMSF plot of 7ng7-MOL000378 complex.
