# Supplementary figures and images for: A Network Pharmacology Prediction and Molecular Docking-Based Strategy to Explore the Potential Pharmacological Mechanism of Astragalus membranaceus for Glioma
Source: Int J Mol Sci. 2023 Nov 14;24(22):16306. doi: 10.3390/ijms242216306 (PMC10671347; doi:10.3390/ijms242216306)

**A**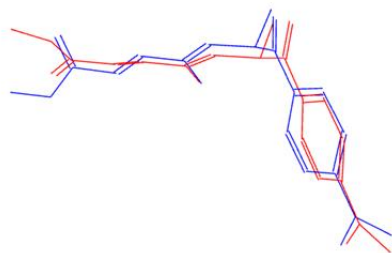**B**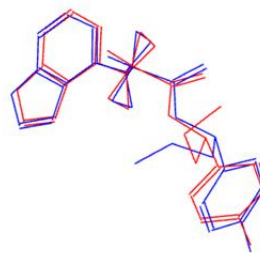**C**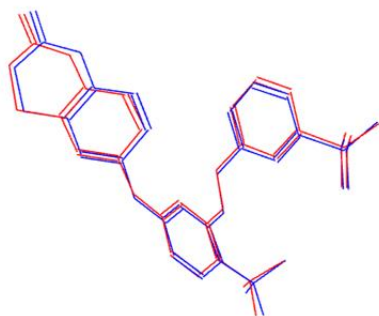**D**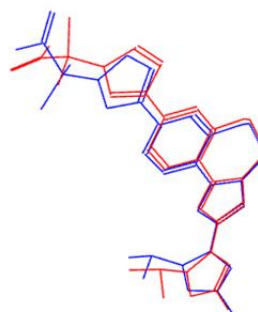

Figure S1. Poses of co-crystallized ligands and redocking ligands. (A) 3C10. (B) 4GV1, (C) 6YOJ. (D) 8EXL.

Supplement: Supplementary file 1 [file ijms-24-16306-s001.zip › Figure S1.Poses of co-crystallized ligands and redocking ligands .pdf]
